# Peripheral Neuropathy in Metabolic Stress

**DOI:** 10.3390/ijms27167301

**Published:** 2026-08-16

**Authors:** Koukou Fu, Jing Yang, Ying Cao

**Affiliations:** School of Life Sciences, Peking University Third Hospital, Peking University, Beijing 100871, China; 2301110504@stu.pku.edu.cn

**Keywords:** pathological axon degeneration, peripheral neuropathy, NAD^+^, SARM1, metabolic organs

## Abstract

It has been broadly recognized that the nervous system is intertwined with various aspects of the body’s metabolism. However, research over the past decade has unexpectedly revealed the involvement of the key metabolic coenzyme nicotinamide adenine dinucleotide (NAD^+^) and its degrading protein sterile alpha and Toll/interleukin-1 receptor motif-containing protein 1 (SARM1) in the central mechanism of pathological axon degeneration, which is the hallmark of peripheral neuropathy. Moreover, the recent implementation of state-of-the-art imaging techniques has achieved the comprehensive assessment of neuroanatomy in major metabolic organs, e.g., liver and adipose tissues, demonstrating that local neural inputs exhibit profound neuropathic changes under metabolic stress, some of which depend on the NAD^+^/SARM1 signal pathway. Here, we summarize current knowledge of the intrinsic link between pathological axon degeneration and metabolic stress and its actions in metabolic organs. In addition, we highlight critical questions regarding this new theme of neurometabolism that call for future research.

## 1. Introduction

The nervous system is intertwined with various aspects of the body’s metabolism, and pathological alterations of such neurometabolic interactions may contribute to the onset or progression of critical human diseases. Of importance, research by our colleagues and us over the past decade has established the central mechanism underlying pathological axon degeneration, which is the hallmark of peripheral neuropathy. In particular, the key metabolic coenzyme nicotinamide adenine dinucleotide (NAD^+^) designates this neuropathic process via its link to a death pathway mediated by sterile alpha and Toll/interleukin-1 receptor motif-containing protein 1 (SARM1). Notably, this NAD^+^/SARM1 signaling pathway is distinct from all the known types of programmed cell death, e.g., apoptosis, necroptosis, or pyroptosis. Moreover, metabolic stress may impinge on the integrity of peripheral neural innervations in major metabolic organs, e.g., liver and adipose tissues. Recent studies by our colleagues and us, empowered by advanced three-dimensional (3D) imaging techniques, have demonstrated that local neural inputs to those organs can exhibit profound peripheral neuropathy. Therefore, the pathophysiological crosstalk of peripheral neuropathy and metabolic stress has emerged as a new theme that increasingly garners research attention in biomedical science. This review aims to summarize current knowledge of such an intrinsic crosstalk between pathological axon degeneration and metabolic stress and to highlight several critical questions for future investigations.

## 2. Metabolic Regulation of Pathological Axon Degeneration

Peripheral neuropathy includes a collection of neurological conditions with damage to sensory, motor, sympathetic, or parasympathetic axonal structures of the peripheral nervous system. Depending on the axonal types involved, it can manifest as sensory loss, neuropathic pain, muscle weakness, or autonomic dysfunction. Damage or breakdown of axons, formally known as pathological axon degeneration, is the central event of peripheral neuropathy, which broadly involves sensory, motor, or autonomic innervations in different parts of the body. Extensive research has documented that this neuropathic process exhibits several key features, including the disruption of axonal transport, deficit of energy homeostasis, overload of intracellular Ca^2+^, and activation of Ca^2+^-dependent calpain proteases [1,2,3,4,5]. Notably, consistent with its distinction from programmed cell death, axonal destruction can occur before or independently of the demise of neuronal cell bodies, which may reside far from innervating tissues, e.g., over 1 m for sensory or motor neurons targeting an adult’s hands or feet. While the classic apoptotic pathway controls the loss of neuronal cells in many neurological conditions, it has a relatively minor role in pathological axon degeneration [6,7,8,9,10]. In addition, evidence has suggested that pathological axon degeneration does not involve other death pathways, e.g., necroptosis, pyroptosis, or ferroptosis [11,12,13,14]. For instance, pyroptosis is a form of programmed cell death involving the cleavage of gasdermin proteins, followed by plasma membrane pore formation, cell swelling, and the release of inflammatory mediators from stressed cells. However, the pyroptotic pathway may not directly contribute to pathological axon degeneration [15,16]. On the other hand, research breakthroughs over the past decade have established the unexpected, essential role of metabolic control in pathological axon degeneration.

### 2.1. WLD^s^ and NMNATs

Research into pathological axon degeneration can be dated back to the British physiologist Augustus Waller, who first reported in 1850 that traumatic injury to peripheral nerves in frogs led to a stereotyped neuropathy [17]. In recognition of this historical observation, pathological axon degeneration is also known as Wallerian degeneration. Modern research into Wallerian degeneration was re-ignited in the 1980s when a group of British neuroscientists serendipitously identified a mutant mouse line, i.e., *Wallerian degeneration slow* (*Wld^s^*), in which the breakdown of traumatically injured axons was delayed for weeks [18]. Detailed studies revealed that *Wld^s^* is the triplication mutation of an 85 kb region on mouse chromosome 4 [19,20]. This 85 kb region originally contains the coding sequences of nicotinamide mononucleotide adenylyltransferase 1 (NMNAT1), retinol binding protein 7, and ubiquitin conjugation factor E4B (UBE4b). Of importance, a mutant protein is generated across the boundary of every two triplicated regions by the in-frame fusion of the N-terminal 70 amino acids of UBE4b with the full-length NMNAT1, which confers axonal protection and thereby has been named the WLD^s^ mutant protein [21]. Research then demonstrated that the WLD^s^ mutant protein does not directly involve the ubiquitin system, as implicated by the partial inclusion of UBE4b. Rather, NMNAT1 catalyzes the final step of synthesizing the key coenzyme NAD^+^ from nicotinamide mononucleotide (NMN) and adenosine triphosphate (ATP), and this enzymatic activity is indispensable for the protective action of WLD^s^ [22]. In support of this link between pathological axon degeneration and NAD^+^ metabolism, axonal NAD^+^ levels precipitate upon various neurological insults, which consequentially causes the blockade of essential steps in glycolysis and the tricarboxylic acid cycle, further resulting in the deficit of axonal ATP levels [23]. Conversely, pharmacological approaches to sustaining NAD^+^ or ATP levels can effectively preserve axonal survival [23,24].

Three isoforms of NMNATs are expressed in mammalian cells, i.e., NMNAT1, NMNAT2, and NMNAT3, with distinct subcellular localizations [25,26]. While NMNAT1 is concentrated within the nucleus, NMNAT2 is present in the cytosol and may be associated with membrane-bound vesicles via protein palmitoylation [27]. NMNAT3 is suggested to reside inside mitochondria, though its precise subcellular localization is still under debate. Among them, NMNAT2 is actively subjected to ubiquitination and degraded by proteasomes in damaged axons, and inhibition of proteasomal activity delays pathological degeneration [28,29]. In addition, genetic deficiency of NMNAT2 in mice exhibits widespread defects in the central and peripheral nervous systems at the embryonic stage, which can be reversed by the WLD^s^ mutant protein [30]. Moreover, loss-of-function mutations of NMNAT2 in humans are associated with severe neurological defects resulting in embryonic lethality or childhood polyneuropathy [31,32]. These findings have demonstrated a vital role of NMNAT2 in maintaining axonal survival, with the WLD^s^ mutant protein functionally replacing the labile endogenous NMNAT2 protein to block the onset of pathological axon degeneration.

### 2.2. SARM1 and NAD^+^ Homeostasis

Genetic screening in the 2010s revealed that the deletion of *Drosophila* sterile alpha and Armadillo motif (dSARM) or its mouse ortholog SARM1 significantly prolongs the survival of damaged axons both in vivo and in vitro to the extent comparable to, if not more robust than, the WLD^s^ mutant protein [33,34]. On the other hand, dSARM or SARM1 appears dispensable for normal neurodevelopment in *Drosophila* or mice, respectively [33,35]. The mammalian SARM1 protein belongs to the Toll/interleukin-1 receptor (TIR) domain-containing adaptor family, which also contains MYD88, TRIF, TRAM, and TIRAP [36]. These other four members have been extensively studied in the field of immunology, e.g., innate immune responses mediated by Toll-like receptors (TLRs). On the contrary, SARM1 expression is enriched in neurons of the central and peripheral nervous systems but highly limited in immune cell types [35]. The SARM1 protein is localized in the cytosol and may be associated with mitochondria or membrane-bound vesicles through its N-terminal sequence [34,37].

Studies surprisingly uncovered that the TIR domain of SARM1 from different species, including fruit flies, fish, mice, and humans, catalyzes the degradation of NAD^+^, producing nicotinamide and adenosine diphosphate ribose (ADPR) or cyclic ADPR (cADPR) [38]. In contrast, the TIR domain derived from other TIR domain-containing proteins in mammals, such as MYD88 and TLR4, does not possess such NAD^+^-degrading ability. The NADase activity of mammalian SARM1 proteins critically depends on a glutamate residue (E642) of the TIR domain, and the E642A mutation abolishes the capacity of SARM1 to trigger pathological axon degeneration [38]. In particular, cADPR produced by active SARM1 has emerged as a novel biomarker for many neurological diseases [39,40,41].

Because the SARM1 protein is consistently expressed in neurons and their axons, its activity must be inhibited under healthy and undamaged conditions. The high-resolution full-length protein structures of SARM1 were obtained by our colleague and us in the 2020s, showing that SARM1 is organized as an octamer via the interaction of its sterile alpha motif (SAM) domain [42,43,44,45,46,47]. Of importance, the NADase activity of the TIR domain is blocked through its binding to the N-terminal Armadillo motif (ARM) domain within the octamer complex. Mutations of several residues along this TIR-ARM interface can significantly boost the NADase activity of SARM1 to the extent that they initiate the degeneration of undamaged axons in cultured neurons. As a caveat, while these reported structures have elucidated the inactive conformation of SARM1, the active state of the protein remains incompletely understood. Although recent studies postulated that the TIR domain needs to dimerize or oligomerize to become activated [44,48,49], a full-length structure of active SARM1 protein is yet to be achieved.

More importantly, research observed that the ARM domain contains a previously unknown pocket for NAD^+^ binding [45,47]. Similar to the mutations that disrupt the TIR-ARM interface, mutations of several critical residues of NAD^+^ binding within the ARM domain dramatically enhance the NADase activity of SARM1, and such dysregulated SARM1 proteins can trigger the degeneration of undamaged axons in cultured neurons, proving that NAD^+^ is not only a substrate of SARM1 but also an allosteric inhibitor of the protein. In support of this notion, detailed biochemical analyses showed that the NADase activity of SARM1 is suppressed by high concentrations of NAD^+^, e.g., over 0.5 mM, but increases as NAD^+^ concentrations decline. Notably, intracellular NAD^+^ levels of mammalian cells are reported to be approximately 0.5 mM [50,51], and it appears conceivable that such high levels of NAD^+^ in the cytosol of healthy axons suffice to inactivate SARM1. On the other hand, interference with NAD^+^ metabolism upon neurological insults, i.e., NMNAT2 depletion, disrupts this NAD^+^-mediated allosteric inhibition, and active SARM1 protein then degrades more NAD^+^ molecules to establish a positive feedback loop leading to axonal death.

**Figure 1:**
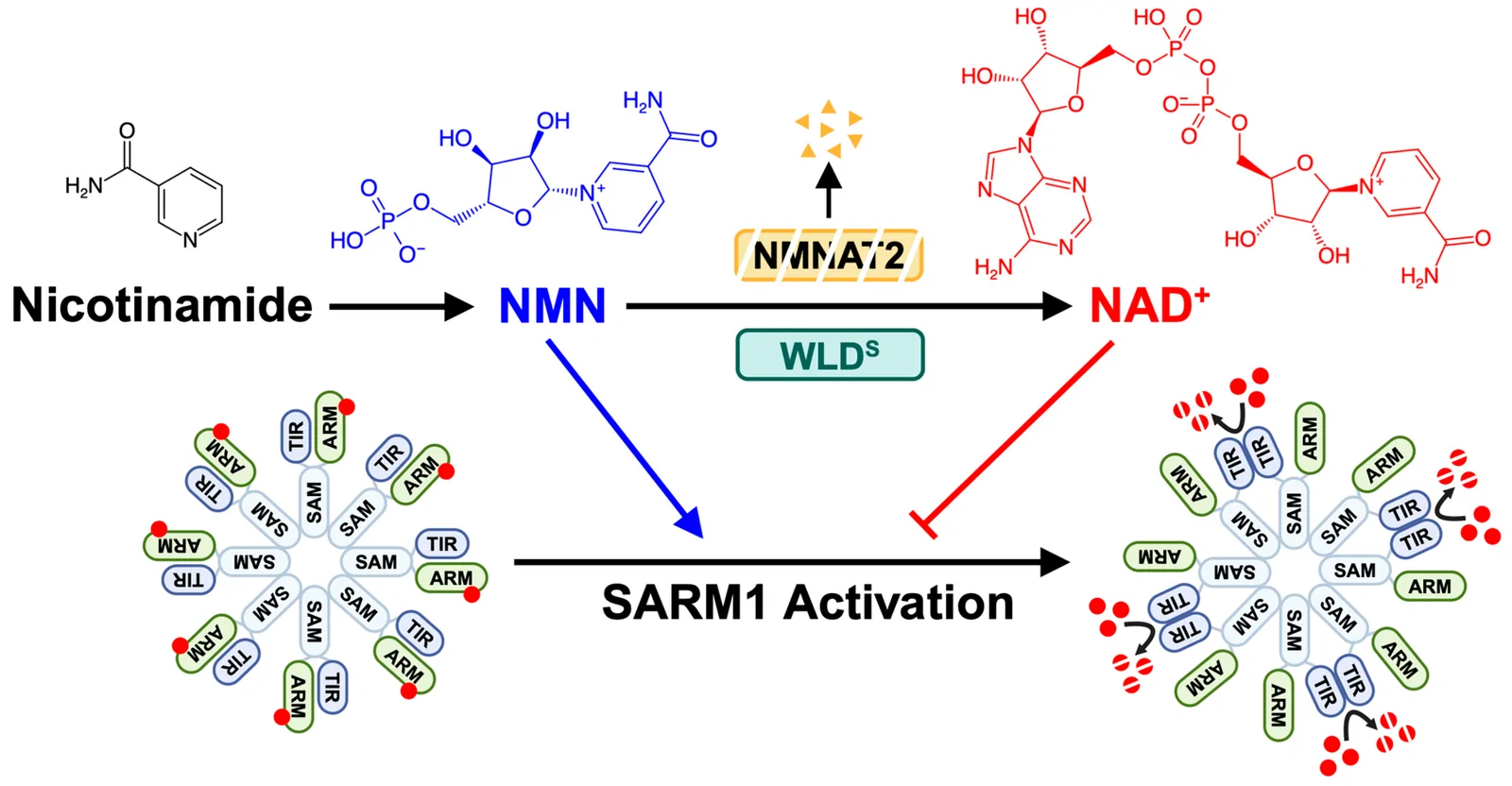
NAD^+^ metabolism regulates SARM1 in pathological axon degeneration. Degradation of nicotinamide mononucleotide adenylyltransferase 2 (NMNAT2) in damaged axons blocks nicotinamide adenine dinucleotide (NAD^+^) synthesis, resulting in an accumulation of nicotinamide mononucleotide (NMN) and a decrease in NAD^+^, which collectively activate the sterile alpha and Toll/interleukin-1 receptor motif-containing protein 1 (SARM1). The active SARM1 then degrades more NAD^+^ molecules, establishing a positive feedback loop of axonal NAD^+^ depletion. This NAD^+^-dependent mechanism results in the downstream energy deficit that triggers pathological axon degeneration. Conversely, expression of the Wallerian degeneration slow (WLD^S^) mutant protein or genetic deletion of SARM1 preserves NAD^+^ metabolism and prevents axonal death. Created in BioRender [60].

Further adding to the complexity, the signaling link between SARM1 and NAD^+^ metabolism extends beyond the NAD^+^ molecule itself. Studies have shown that the NADase activity of SARM1 is also influenced by the two NAD^+^ metabolic precursors, i.e., nicotinamide and NMN. Nicotinamide can inhibit the NADase activity of the TIR domain, though it requires high concentrations, implicating a mechanism of end-product inhibition [38]. On the contrary, the NMNAT2 depletion in damaged axons blocks the conversion of NMN to NAD^+^, and axonal NMN levels accumulate as a result [52]. Significantly, NMN is an activator of the NADase activity of SARM1 and likely contributes to the initiation of pathological axon degeneration [53,54,55]. Moreover, NMN can fit into the NAD^+^-binding pocket of the ARM domain and likely disrupt the NAD^+^-mediated allosteric inhibition of SARM1, though a high-resolution structure of the full-length SARM1 protein in the active conformation with NMN still awaits future research. Together, a paradigm has emerged that the homeostasis of NAD^+^ metabolism designates the NADase activity of SARM1, acting as the central control of pathological axonal degeneration (Figure 1).

In accordance with the key role of metabolic regulation of pathological axon degeneration, mitochondrial dysfunction may act as an upstream trigger of the SARM1-dependent pathway. For instance, disruption of the mitochondrial membrane potential in mouse sympathetic axons causes NMNAT2 depletion, thereby increasing the NMN/NAD^+^ ratio to activate SARM1 [56]. Mitochondrial depolarization and the associated accumulation of reactive oxygen species could also induce the SARM1-dependent degeneration of sensory axons [57]. Moreover, autophagy, a lysosome-dependent process that removes misfolded proteins and dysfunctional organelles such as mitochondria, plays an essential role in maintaining axonal homeostasis [58,59]. For example, genetic loss of an essential autophagy protein autophagy-related 7 (ATG7) in mouse sensory neurons could trigger axonal degeneration before neuronal cell death [59]. Of importance, SARM1 was activated in those mouse neurons, whereas expression of a dominant-negative SARM1 protein prevented such sensory neuropathy [59]. These findings have implicated that autophagy is linked to SARM1-dependent pathological axon degeneration, although the detailed underlying mechanism remains unclear.

## 3. Peripheral Neuropathy in Metabolic Organs

It has been broadly recognized that the nervous system regulates various aspects of the body’s metabolism, e.g., food intake, nutrient absorption, and energy expenditure, dysfunction of which leads to diabetes, fatty liver, obesity, and other metabolic-related diseases [61,62,63,64]. At the same time, metabolic stress can reciprocally impinge on the integrity of the nervous system. For instance, peripheral sensory axons in the feet, legs, and hands of diabetic patients often undergo pathological damage, formally known as diabetic peripheral neuropathy, resulting in chronic pain and numbness [65,66,67]. These neuropathic events involve the degeneration of small sensory fibers, including thinly myelinated Aδ fibers and unmyelinated C fibers [68]. Such axonal loss can be assessed by measuring intraepidermal nerve fiber density (IENFD) in skin biopsies with immunostaining for neural markers such as protein gene product 9.5 (PGP9.5). Significantly, diabetic peripheral neuropathy represents one of the most common complications of diabetes and depends on the NAD^+^/SARM1 signal pathway in the mouse disease models [69,70].

**Figure 2:**
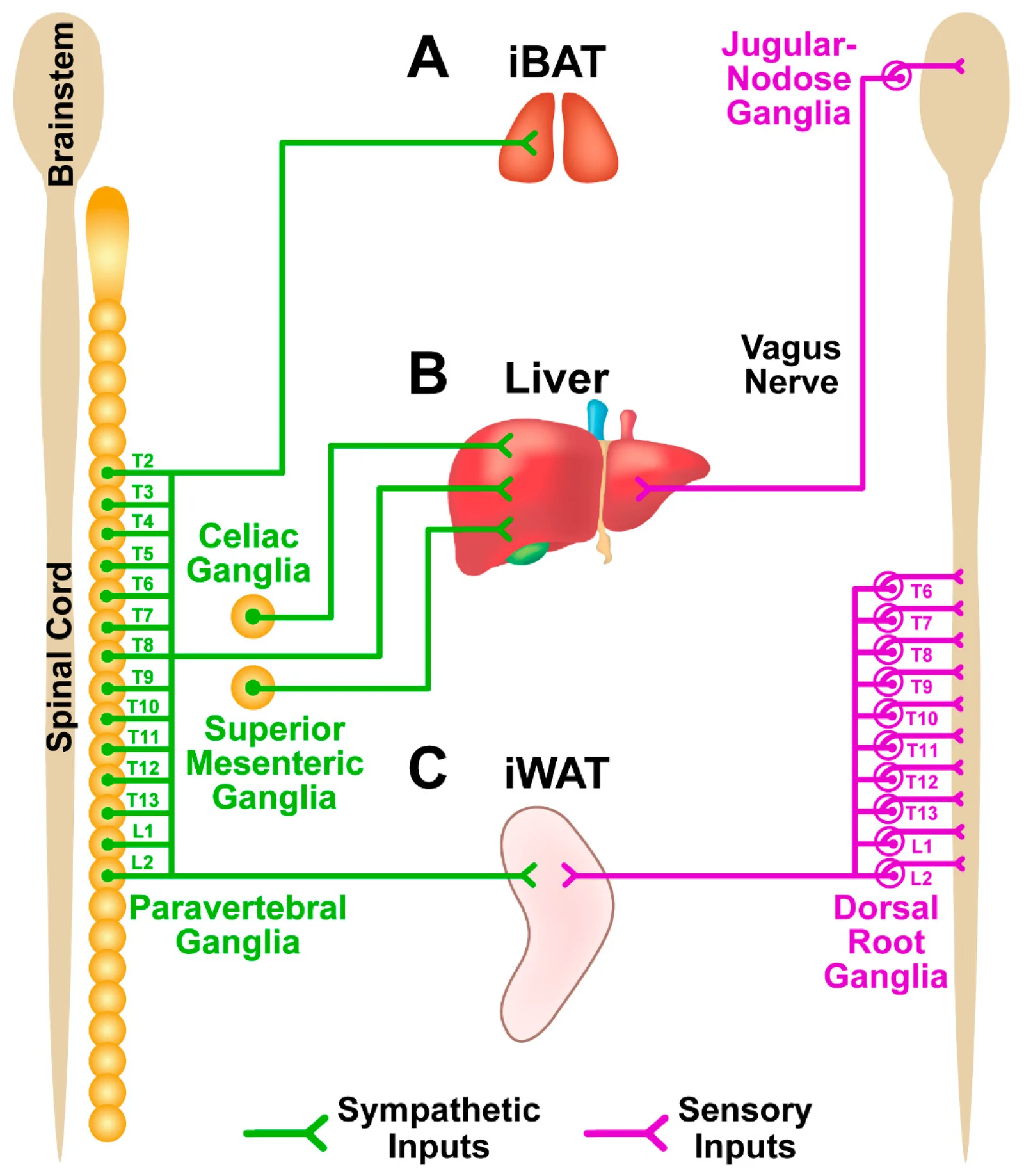
Neuroanatomy of major metabolic organs. (**A**) Interscapular brown adipose tissues (iBATs) of rodents are innervated by sympathetic inputs from the thoracic paravertebral ganglia. There are likely no parasympathetic or sensory innervations in iBATs. (**B**) The liver of rodents or humans has sympathetic inputs from the celiac ganglia, superior mesenteric ganglia, and thoracic paravertebral ganglia. Also, the liver receives sensory axons from the jugular-nodose ganglia via the vagus nerve. There is a minor presence of parasympathetic axons in the liver parenchyma. (**C**) Inguinal white adipose tissues (iWATs) of rodents or humans have sympathetic inputs from the thoracic and lumbar paravertebral ganglia. Also, iWATs receive sensory axons from the thoracic and lumbar dorsal root ganglia. There are likely no parasympathetic innervations in iWATs. T, thoracic; L, lumbar.

Notably, major metabolic organs of the body, e.g., liver and adipose tissues, receive autonomic or sensory inputs (Figure 2). In particular, postganglionic sympathetic neurons express tyrosine hydroxylase (TH) and dopamine β-hydroxylase (DBH), the two enzymes essential for synthesizing the neurotransmitter norepinephrine [71,72]. Meanwhile, postganglionic parasympathetic neurons are characterized by choline acetyltransferase (ChAT), the central enzyme in producing the neurotransmitter acetylcholine, and vesicular acetylcholine transporter (VAChT), the protein responsible for loading acetylcholine from the cytosol into synaptic vesicles [71,73]. In addition, while diverse subtypes exist in vagal sensory neurons [74,75,76] or spinal sensory neurons [77,78,79,80], sensory inputs generally release the neurotransmitter glutamate or neuropeptides, such as calcitonin gene-related peptide (CGRP) and substance P (SP) [81,82,83]. However, although autonomic or sensory inputs in the liver or adipose tissues have long been documented, conventional immunohistochemical analyses of tissue sections have intrinsic limitations in visualizing the three-dimensional (3D) distribution of neural structures. Over the past decade, studies by our colleagues and us have exploited state-of-the-art imaging techniques that combine whole-tissue immunolabeling, optical clearing, and multiphoton or lightsheet microscopy, which enable the comprehensive assessment of neuroanatomy in intact, unsectioned metabolic organs and their neuropathic alterations under disease conditions.

### 3.1. Liver

**Figure 3:**
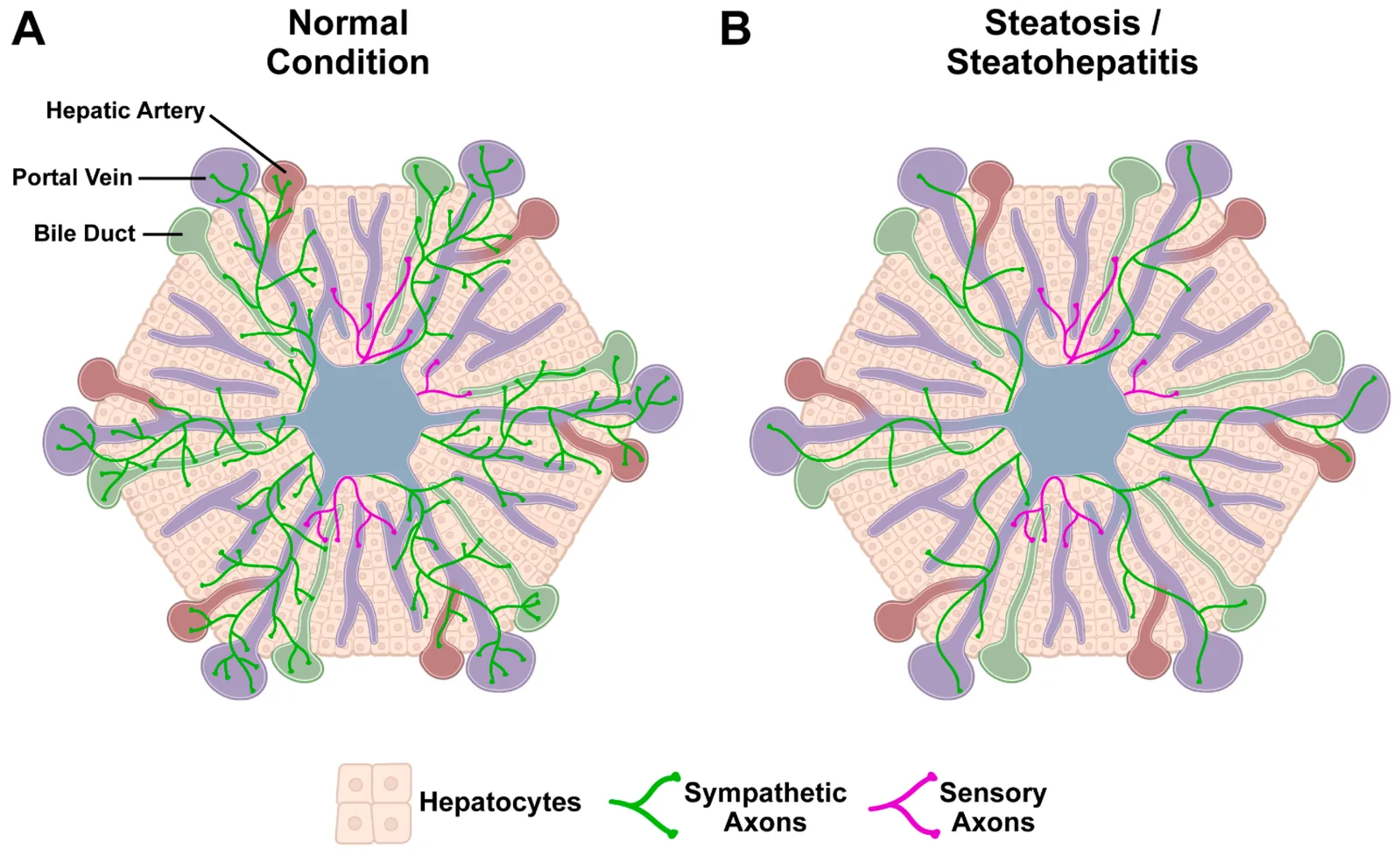
Neuropathic changes in the liver under metabolic stress. (**A**) In the normal condition, sympathetic and sensory axons in the liver of rodents or humans project along the portal triad, i.e., portal vein, hepatic artery, and bile duct. (**B**) In steatosis or steatohepatitis, neuropathic loss of sympathetic inputs occurs in the liver, while sensory axons may exhibit no apparent change. Sympathetic axons are in green, and sensory axons are in magenta.

Sympathetic inputs in the liver have been observed for decades [84,85]. Building upon those classic studies, advanced 3D imaging research has recently charted the complete anatomy of sympathetic distribution in the liver of mice, macaques, and humans [86,87]. In particular, TH-positive sympathetic fibers primarily project along the portal triad, i.e., portal vein, hepatic artery, and bile duct, but are mostly absent around the hepatic vein (Figure 3). Of importance, these 3D imaging analyses have revealed, for the first time, a profound loss of sympathetic axons in the liver tissues of mice under metabolic stress, such as nonalcoholic fatty liver disease (NAFLD) induced by a high-fat or high-fat/high-sucrose diet, or with leptin deficiency, i.e., *ob*/*ob* and *db*/*db* [86,87]. Also, such sympathetic neuropathy can be similarly observed in the liver tissues of patients with steatosis or steatohepatitis, positively correlating with the severity of NAFLD pathology [86]. Research further showed that the proinflammatory cytokine TNFα, derived from macrophages or Kupffer cells in metabolically stressed liver tissues, directly induces the degeneration of sympathetic axons in a SARM1-dependent manner. Accordingly, genetic deletion of SARM1 effectively protects sympathetic axons in the liver tissues of mice with NAFLD induced by a high-fat diet, and this preservation of sympathetic inputs mitigates tissue inflammation and improves the insulin response of hepatocytes and overall glucose metabolism of the mice [87]. As a caveat, the detailed mechanism linking TNFα to NAD^+^ metabolism and SARM1 activation in sympathetic axons remains uncharacterized. In addition, a portion of sympathetic inputs were still lost in the liver tissues of SARM1-deficient mice under the NAFLD condition, implicating the involvement of other death mechanism(s) in this scenario [87].

In contrast to sympathetic innervation, though early studies reported hepatic parasympathetic inputs [88,89,90], advanced 3D imaging analyses have demonstrated the absence of VAChT-positive parasympathetic axons in the liver tissues of mice, macaques, and humans [86,87]. Also, genetic labeling with a ChAT-reporter mouse line revealed few parasympathetic fibers in the liver parenchyma [87]. These results have indicated that at least in those examined species, the liver has a minor presence of parasympathetic inputs, which helps clarify an important neuroanatomical feature of this metabolic organ.

Peptidergic sensory axons innervating the liver have long been documented [91,92,93,94]. 3D imaging has confirmed that CGRP-positive or SP-positive sensory axons project along the portal triad in a pattern similar to, but at a lower density than, that of sympathetic fibers in the liver tissues of mice or humans [86,95,96]. Intriguingly, contrary to sympathetic neuropathy observed in the mouse models of steatosis or steatohepatitis, those CGRP-positive sensory inputs remained stable over the disease course [86]. Whether this resistance of peptidergic sensory axons to degeneration may be due to a lower or absent expression of the TNFα receptor or its downstream components linking to SARM1 activation is an important question for future research. In addition, whether other subtypes of sensory axons, e.g., glutamatergic, exist in the liver and whether they may exhibit any neuropathic change under metabolic stress is currently unknown.

### 3.2. Brown Adipose Tissues

**Figure 4:**
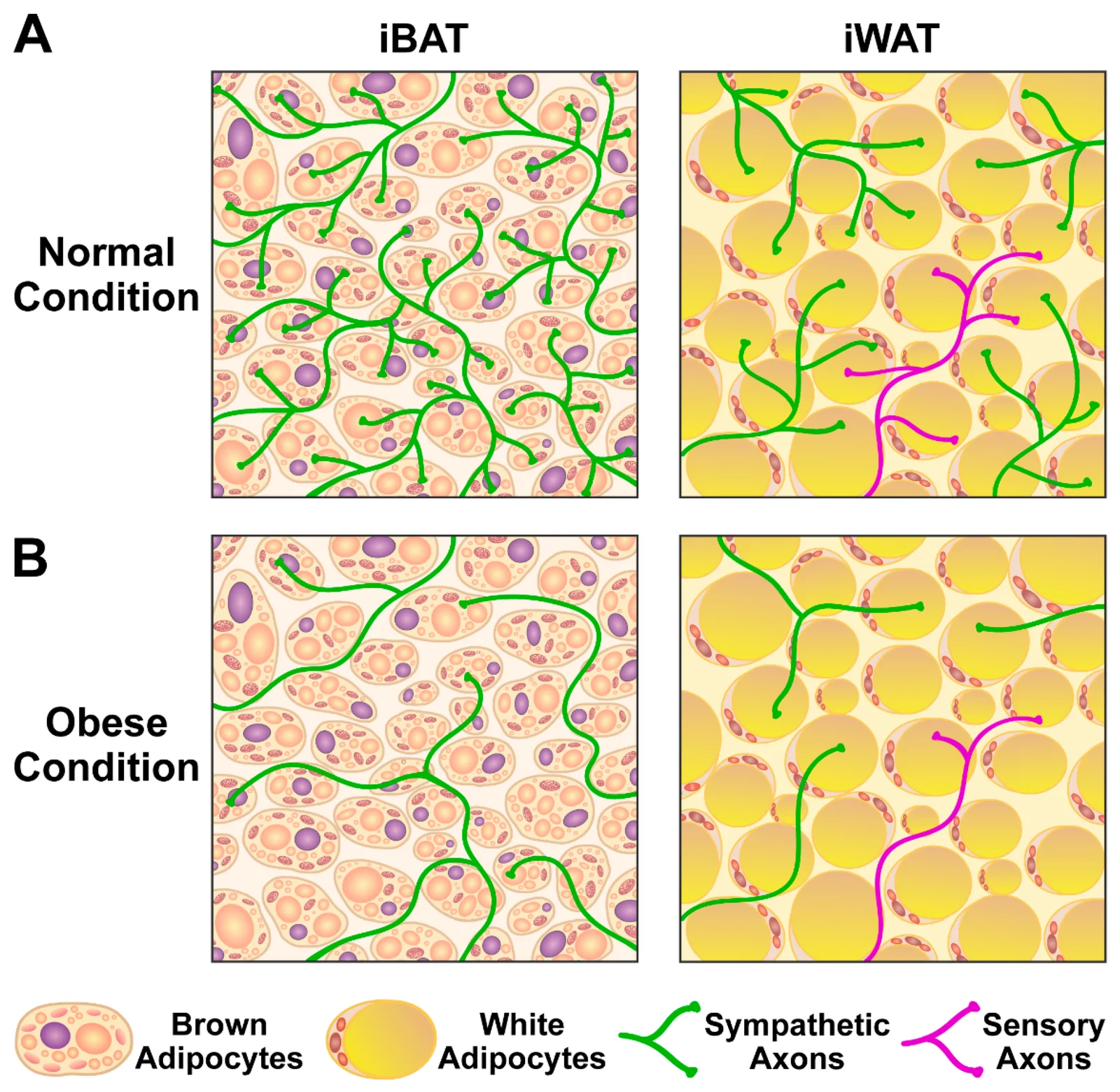
Neuropathy in brown and white adipose tissues under metabolic stress. (**A**) In the normal condition, sympathetic inputs are in direct contact with adipocytes in interscapular brown adipose tissues (iBATs, **left panel**) of rodents or inguinal white adipose tissues (iWATs, **right panel**) of rodents or humans. Sensory inputs are also present in iWATs. (**B**) In obese conditions, neuropathic loss of sympathetic inputs occurs in iBATs and iWATs of rodents or humans. Whether sensory axons in iWATs may undergo neuropathic change under metabolic stress is currently unknown. Sympathetic axons are in green, and sensory axons are in magenta.

Sympathetic action in brown adipose tissues (BATs) has been well established, which is essential for non-shivering thermogenesis [97,98]. Conventional immunohistochemical analyses revealed sympathetic fibers projecting along vasculatures and in direct contact with brown adipocytes in rodent BATs [99,100,101]. Advanced 3D imaging studies have enabled more comprehensive visualization of TH-positive sympathetic axons in rodent BATs, e.g., interscapular brown adipose tissues (iBATs) (Figure 4) [102,103,104,105]. On the other hand, because of the overall lack of BATs in adult humans, anatomical evidence for sympathetic inputs is relatively limited, and only recent studies have reported TH-positive sympathetic fibers in the supraclavicular BAT depot [106,107]. Of importance, sympathetic inputs to BATs can undergo neuropathic damage in metabolic disease conditions. For instance, early reports showed reduced sympathetic densities in iBATs of *ob*/*ob* mice or high-fat diet-fed rats [108,109,110]. 3D imaging analyses have further confirmed a profound loss of TH-positive sympathetic fibers in iBATs of mice fed with a high-fat diet or with leptin deficiency, i.e., *ob*/*ob* or *db*/*db* mice [102,103]. Because the local sympathetic signal is the principal driver of the thermogenesis of BATs, its neuropathy can directly contribute to the impairment of thermogenic activity and energy expenditure in those mouse models and may also be relevant to disrupted metabolic homeostasis in patients with obesity [111]. However, the detailed mechanism responsible for sympathetic neuropathy in BATs remains mostly undefined. Though evidence has suggested that tissue inflammation under metabolic stress, particularly proinflammatory macrophages and their derived cytokines such as TNFα, may influence the establishment or maintenance of sympathetic structures [112,113,114,115], it is still unclear whether this neuropathic event depends on the NAD^+^/SARM1 signal pathway or other death programs.

In contrast to the dense presence of sympathetic inputs, VAChT-positive parasympathetic fibers are not detected in interscapular, cervical, and perirenal BATs of rodents, whereas their presence was reported in mediastinal BAT, a minor BAT depot [116]. Meanwhile, VAChT-positive parasympathetic inputs were only observed in human thoracic BAT but not in other BATs [117]. Therefore, it appears to be a general theme that parasympathetic inputs are rare, if not absent, in BATs. Understandably, due to their highly limited distribution, whether those parasympathetic structures may undergo any neuropathic change under disease conditions is entirely unknown.

Studies have confirmed that rodent BATs receive peptidergic sensory inputs. In particular, CGRP-positive or SP-positive sensory axons can be detected in iBATs and perirenal BAT depots [118,119,120,121]. However, whether they may be affected by metabolic stress is unclear. In addition, whether other subtypes of sensory inputs may exist in rodent BATs awaits more detailed examination. Furthermore, given the sparsity of human BATs, whether they contain any sensory fibers remains to be investigated.

### 3.3. White Adipose Tissues

Although it has long been observed that the local sympathetic action can trigger the lipolysis of white adipose tissues (WATs) [122], conventional immunohistochemical studies showed a low density of sympathetic inputs that mainly project along blood vessels in WATs but sparsely contact white adipocytes [123,124,125]. This led to a canonical view that the metabolism of adipocytes would be primarily controlled by circulating nutrients or hormones, and as a result, the neuroanatomy of WATs and its pathophysiological relevance had been historically underappreciated [126,127]. The implementation of 3D imaging techniques has revealed abundant TH-positive sympathetic fibers in direct contact with adipocytes in inguinal WATs (iWATs) or epididymal WATs of mice [102,128] (Figure 4). Also, 3D imaging has visualized TH-positive sympathetic inputs in subcutaneous or omental WATs of humans [129,130]. Of more importance, similar to that aforementioned in BATs, there is a dramatic loss of sympathetic fibers in WATs of mice fed with a high-fat diet or with leptin deficiency, i.e., *ob*/*ob* or *db*/*db* mice [102,103,131]. Also, reduced sympathetic densities are observed in the subcutaneous WATs of patients with obesity [132]. Because sympathetic signal is essential to promote the lipolysis of white adipocytes [133], such peripheral neuropathy exaggerates lipid accumulation in WATs, a central pathological feature underlying the disease progression of obesity. In addition, research has demonstrated that the local sympathetic signal is critical for the appearance of beige adipocytes in WATs, i.e., the beiging process, under prolonged cold exposure [102,128], and their pathological loss impairs the energy expenditure of mice under metabolic stress. However, the mechanism underlying sympathetic neuropathy in WATs is still uncharted. In particular, it is conceivable that this neuropathic event may be triggered by the inflammation of WATs, which commonly occurs in obesity, and may depend on the NAD^+^/SARM1 pathway as observed in the context of metabolically stressed liver tissues.

In contrast to sympathetic inputs, studies appear to reach a consensus that ChAT-positive or VAChT-positive parasympathetic fibers are not detected in rodent or human WATs [134,135,136], albeit a couple of early studies had implicated such a presence [137,138].

Similar to BATs, CGRP-positive peptidergic sensory axons have been documented in rodent iWATs [139]. Also, recent studies reported PIEZO2-positive mechanosensory inputs in mouse iWATs [140,141]. Such direct connections between iWATs and sensory neurons of dorsal root ganglia are supported by viral or genetic tracing in mice combined with advanced 3D imaging [142]. However, whether comparable subtypes of sensory inputs exist in human WATs is entirely unknown. In addition, whether specific subtypes of sensory axons may be affected by metabolic stress is yet to be examined.

## 4. Conclusions

We have witnessed significant advances in understanding the intrinsic link between pathological axon degeneration and metabolic stress, which has been increasingly recognized as a new frontier of biomedical research over the past decade. However, several key questions call for future research efforts.

(1) Neuroanatomy of sensory inputs in metabolic organs. While 3D imaging analyses have achieved an unprecedented, comprehensive anatomy of sympathetic or parasympathetic inputs in the liver and adipose tissues, sensory axons in those organs remain much less well characterized. Of importance, diverse subtypes of vagal sensory neurons [74,75,76] or spinal sensory neurons [77,78,79,80] are known to exist, posing a key challenge to their precise assessment under physiological or pathological conditions. Notably, diabetic peripheral neuropathy, i.e., the destruction of peripheral sensory axons in the limbs of diabetic patients, has long been documented and is dependent on the NAD^+^/SARM1 pathway [69,70]. In contrast, whether there is neuropathic loss of any specific subtype of sensory inputs in those metabolic organs is currently unknown. Moreover, it is plausible that the subtypes of sensory inputs in different depots of adipose tissues of the body, e.g., visceral WATs *versus* inguinal WATs, vary significantly in receiving the axonal projections of vagal or spinal neurons, further complicating their anatomical analyses in metabolic homeostasis or stress.

(2) Mechanisms of peripheral neuropathy in metabolic organs. Research has shown that the proinflammatory cytokine TNFα can cause sympathetic neuropathy in the liver, which relies on the NAD^+^/SARM1 signal pathway [87,143]. However, whether this SARM1-dependent mechanism may designate sympathetic neuropathy in brown or white adipose tissues is still unclear. In addition, studies reported that administration of the recombinant leptin protein is sufficient to reverse sympathetic neuropathy in the liver and adipose tissues of *ob/ob* mice [87,103]. How this leptin action intersects with the NAD^+^/SARM1 pathway remains an important question.

(3) Disease relevance of peripheral neuropathy in metabolic organs. Recent studies have begun to elucidate that peripheral neuropathy in the liver or adipose tissues can impair insulin response, glucose metabolism, or energy expenditure [87,112,133,139,144,145,146,147]. Such pathological effects reflect the direct action of neurotransmitters on metabolism-related cell types, e.g., hepatocytes and adipocytes. At the same time, neurotransmitters may also influence non-metabolic cell types, such as immune cells, endothelial cells, and other stromal cells of those organs. Therefore, the overall outcome of a neuropathic event integrating neural actions across various cell types within a metabolic organ warrants more detailed investigations.

(4) Therapeutic targeting of SARM1 in peripheral neuropathy. Research over the past decades has established SARM1 and related NAD^+^ metabolism as promising therapeutic targets for treating various neurological diseases. Indeed, small-molecule inhibitors aiming at the catalytic TIR domain or the allosteric ARM domain of human SARM1 protein have been developed and proven to be effective in mitigating peripheral neuropathy in mouse disease models [48,148,149,150,151]. Moreover, with advances in artificial intelligence (AI) technologies, it has become exciting that AI-assisted discovery and development of small-molecule inhibitors or other antagonistic modalities of SARM1 may accelerate the treatment of important neurological conditions, including peripheral neuropathy [152,153].

In sum, we have here reviewed current knowledge of the crosstalk between pathological axon degeneration and metabolic stress and their pathophysiological roles in major metabolic organs. Research efforts into this neurometabolic frontier hold promise of identifying novel entry points for diagnostic or therapeutic applications against human metabolic diseases.

## Data Availability

No new data were created or analyzed in this study. Data sharing is not applicable to this article.

## References

[1] Coleman M.P., Höke A. (2020). Programmed axon degeneration: From mouse to mechanism to medicine. Nat. Rev. Neurosci..

[2] DiAntonio A. (2019). Axon degeneration: Mechanistic insights lead to therapeutic opportunities for the prevention and treatment of peripheral neuropathy. Pain.

[3] Neukomm L.J., Freeman M.R. (2014). Diverse cellular and molecular modes of axon degeneration. Trends Cell Biol..

[4] Wang J.T., Medress Z.A., Barres B.A. (2012). Axon degeneration: Molecular mechanisms of a self-destruction pathway. J. Cell Biol..

[5] Loreto A., Neukomm L.J. (2026). Programmed axon degeneration: Mechanism, inhibition and therapeutic potential. Nat. Rev. Neurosci..

[6] Chiesa R., Piccardo P., Dossena S., Nowoslawski L., Roth K.A., Ghetti B., Harris D.A. (2005). Bax deletion prevents neuronal loss but not neurological symptoms in a transgenic model of inherited prion disease. Proc. Natl. Acad. Sci. USA.

[7] Gould T.W., Buss R.R., Vinsant S., Prevette D., Sun W., Knudson C.M., Milligan C.E., Oppenheim R.W. (2006). Complete dissociation of motor neuron death from motor dysfunction by Bax deletion in a mouse model of ALS. J. Neurosci..

[8] Libby R.T., Li Y., Savinova O.V., Barter J., Smith R.S., Nickells R.W., John S.W. (2005). Susceptibility to neurodegeneration in a glaucoma is modified by Bax gene dosage. PLoS Genet..

[9] Whitmore A.V., Lindsten T., Raff M.C., Thompson C.B. (2003). The proapoptotic proteins Bax and Bak are not involved in Wallerian degeneration. Cell Death Differ..

[10] Simon D.J., Weimer R.M., McLaughlin T., Kallop D., Stanger K., Yang J., O’Leary D.D., Hannoush R.N., Tessier-Lavigne M. (2012). A caspase cascade regulating developmental axon degeneration. J. Neurosci..

[11] Cao Y., Wang Y., Yang J. (2022). NAD(+)-dependent mechanism of pathological axon degeneration. Cell Insight.

[12] Conforti L., Gilley J., Coleman M.P. (2014). Wallerian degeneration: An emerging axon death pathway linking injury and disease. Nat. Rev. Neurosci..

[13] Geden M.J., Deshmukh M. (2016). Axon degeneration: Context defines distinct pathways. Curr. Opin. Neurobiol..

[14] Gerdts J., Summers D.W., Milbrandt J., DiAntonio A. (2016). Axon Self-Destruction: New Links among SARM1, MAPKs, and NAD+ Metabolism. Neuron.

[15] Broz P., Pelegrín P., Shao F. (2020). The gasdermins, a protein family executing cell death and inflammation. Nat. Rev. Immunol..

[16] Shi J., Zhao Y., Wang K., Shi X., Wang Y., Huang H., Zhuang Y., Cai T., Wang F., Shao F. (2015). Cleavage of GSDMD by inflammatory caspases determines pyroptotic cell death. Nature.

[17] Waller A. (1850). Experiments on the Section of the Glossopharyngeal and Hypoglossal Nerves of the Frog, and Observations of the Alterations Produced Thereby in the Structure of Their Primitive Fibres. Philos. Trans. R. Soc. Lond..

[18] Lunn E.R., Perry V.H., Brown M.C., Rosen H., Gordon S. (1989). Absence of Wallerian Degeneration does not Hinder Regeneration in Peripheral Nerve. Eur. J. Neurosci..

[19] Coleman M.P., Conforti L., Buckmaster E.A., Tarlton A., Ewing R.M., Brown M.C., Lyon M.F., Perry V.H. (1998). An 85-kb tandem triplication in the slow Wallerian degeneration (Wlds) mouse. Proc. Natl. Acad. Sci. USA.

[20] Conforti L., Tarlton A., Mack T.G., Mi W., Buckmaster E.A., Wagner D., Perry V.H., Coleman M.P. (2000). A Ufd2/D4Cole1e chimeric protein and overexpression of Rbp7 in the slow Wallerian degeneration (WldS) mouse. Proc. Natl. Acad. Sci. USA.

[21] Mack T.G., Reiner M., Beirowski B., Mi W., Emanuelli M., Wagner D., Thomson D., Gillingwater T., Court F., Conforti L. (2001). Wallerian degeneration of injured axons and synapses is delayed by a Ube4b/Nmnat chimeric gene. Nat. Neurosci..

[22] Araki T., Sasaki Y., Milbrandt J. (2004). Increased nuclear NAD biosynthesis and SIRT1 activation prevent axonal degeneration. Science.

[23] Wang J., Zhai Q., Chen Y., Lin E., Gu W., McBurney M.W., He Z. (2005). A local mechanism mediates NAD-dependent protection of axon degeneration. J. Cell Biol..

[24] Yang J., Wu Z., Renier N., Simon D.J., Uryu K., Park D.S., Greer P.A., Tournier C., Davis R.J., Tessier-Lavigne M. (2015). Pathological axonal death through a MAPK cascade that triggers a local energy deficit. Cell.

[25] Belenky P., Bogan K.L., Brenner C. (2007). NAD+ metabolism in health and disease. Trends Biochem. Sci..

[26] Cambronne X.A., Kraus W.L. (2020). Location, Location, Location: Compartmentalization of NAD(+) Synthesis and Functions in Mammalian Cells. Trends Biochem. Sci..

[27] Lau C., Dölle C., Gossmann T.I., Agledal L., Niere M., Ziegler M. (2010). Isoform-specific targeting and interaction domains in human nicotinamide mononucleotide adenylyltransferases. J. Biol. Chem..

[28] Gilley J., Coleman M.P. (2010). Endogenous Nmnat2 is an essential survival factor for maintenance of healthy axons. PLoS Biol..

[29] Zhai Q., Wang J., Kim A., Liu Q., Watts R., Hoopfer E., Mitchison T., Luo L., He Z. (2003). Involvement of the ubiquitin-proteasome system in the early stages of wallerian degeneration. Neuron.

[30] Gilley J., Adalbert R., Yu G., Coleman M.P. (2013). Rescue of peripheral and CNS axon defects in mice lacking NMNAT2. J. Neurosci..

[31] Huppke P., Wegener E., Gilley J., Angeletti C., Kurth I., Drenth J.P.H., Stadelmann C., Barrantes-Freer A., Brück W., Thiele H. (2019). Homozygous NMNAT2 mutation in sisters with polyneuropathy and erythromelalgia. Exp. Neurol..

[32] Lukacs M., Gilley J., Zhu Y., Orsomando G., Angeletti C., Liu J., Yang X., Park J., Hopkin R.J., Coleman M.P. (2019). Severe biallelic loss-of-function mutations in nicotinamide mononucleotide adenylyltransferase 2 (NMNAT2) in two fetuses with fetal akinesia deformation sequence. Exp. Neurol..

[33] Osterloh J.M., Yang J., Rooney T.M., Fox A.N., Adalbert R., Powell E.H., Sheehan A.E., Avery M.A., Hackett R., Logan M.A. (2012). dSarm/Sarm1 is required for activation of an injury-induced axon death pathway. Science.

[34] Gerdts J., Summers D.W., Sasaki Y., DiAntonio A., Milbrandt J. (2013). Sarm1-mediated axon degeneration requires both SAM and TIR interactions. J. Neurosci..

[35] Kim Y., Zhou P., Qian L., Chuang J.Z., Lee J., Li C., Iadecola C., Nathan C., Ding A. (2007). MyD88-5 links mitochondria, microtubules, and JNK3 in neurons and regulates neuronal survival. J. Exp. Med..

[36] O’Neill L.A., Bowie A.G. (2007). The family of five: TIR-domain-containing adaptors in Toll-like receptor signalling. Nat. Rev. Immunol..

[37] Panneerselvam P., Singh L.P., Ho B., Chen J., Ding J.L. (2012). Targeting of pro-apoptotic TLR adaptor SARM to mitochondria: Definition of the critical region and residues in the signal sequence. Biochem. J..

[38] Essuman K., Summers D.W., Sasaki Y., Mao X., DiAntonio A., Milbrandt J. (2017). The SARM1 Toll/Interleukin-1 Receptor Domain Possesses Intrinsic NAD(+) Cleavage Activity that Promotes Pathological Axonal Degeneration. Neuron.

[39] Sasaki Y., Engber T.M., Hughes R.O., Figley M.D., Wu T., Bosanac T., Devraj R., Milbrandt J., Krauss R., DiAntonio A. (2020). cADPR is a gene dosage-sensitive biomarker of SARM1 activity in healthy, compromised, and degenerating axons. Exp. Neurol..

[40] Waller T.J., Collins C.A. (2022). Multifaceted roles of SARM1 in axon degeneration and signaling. Front. Cell Neurosci..

[41] McGuinness H.Y., Gu W., Shi Y., Kobe B., Ve T. (2024). SARM1-Dependent Axon Degeneration: Nucleotide Signaling, Neurodegenerative Disorders, Toxicity, and Therapeutic Opportunities. Neuroscientist.

[42] Bratkowski M., Xie T., Thayer D.A., Lad S., Mathur P., Yang Y.S., Danko G., Burdett T.C., Danao J., Cantor A. (2020). Structural and Mechanistic Regulation of the Pro-degenerative NAD Hydrolase SARM1. Cell Rep..

[43] Figley M.D., Gu W., Nanson J.D., Shi Y., Sasaki Y., Cunnea K., Malde A.K., Jia X., Luo Z., Saikot F.K. (2021). SARM1 is a metabolic sensor activated by an increased NMN/NAD(+) ratio to trigger axon degeneration. Neuron.

[44] Horsefield S., Burdett H., Zhang X., Manik M.K., Shi Y., Chen J., Qi T., Gilley J., Lai J.S., Rank M.X. (2019). NAD(+) cleavage activity by animal and plant TIR domains in cell death pathways. Science.

[45] Jiang Y., Liu T., Lee C.H., Chang Q., Yang J., Zhang Z. (2020). The NAD(+)-mediated self-inhibition mechanism of pro-neurodegenerative SARM1. Nature.

[46] Shen C., Vohra M., Zhang P., Mao X., Figley M.D., Zhu J., Sasaki Y., Wu H., DiAntonio A., Milbrandt J. (2021). Multiple domain interfaces mediate SARM1 autoinhibition. Proc. Natl. Acad. Sci. USA.

[47] Sporny M., Guez-Haddad J., Khazma T., Yaron A., Dessau M., Shkolnisky Y., Mim C., Isupov M.N., Zalk R., Hons M. (2020). Structural basis for SARM1 inhibition and activation under energetic stress. eLife.

[48] Shi Y., Kerry P.S., Nanson J.D., Bosanac T., Sasaki Y., Krauss R., Saikot F.K., Adams S.E., Mosaiab T., Masic V. (2022). Structural basis of SARM1 activation, substrate recognition, and inhibition by small molecules. Mol. Cell.

[49] Summers D.W., Gibson D.A., DiAntonio A., Milbrandt J. (2016). SARM1-specific motifs in the TIR domain enable NAD+ loss and regulate injury-induced SARM1 activation. Proc. Natl. Acad. Sci. USA.

[50] Yang H., Yang T., Baur J.A., Perez E., Matsui T., Carmona J.J., Lamming D.W., Souza-Pinto N.C., Bohr V.A., Rosenzweig A. (2007). Nutrient-sensitive mitochondrial NAD+ levels dictate cell survival. Cell.

[51] Yang Y., Mohammed F.S., Zhang N., Sauve A.A. (2019). Dihydronicotinamide riboside is a potent NAD(+) concentration enhancer in vitro and in vivo. J. Biol. Chem..

[52] Di Stefano M., Nascimento-Ferreira I., Orsomando G., Mori V., Gilley J., Brown R., Janeckova L., Vargas M.E., Worrell L.A., Loreto A. (2015). A rise in NAD precursor nicotinamide mononucleotide (NMN) after injury promotes axon degeneration. Cell Death Differ..

[53] Angeletti C., Amici A., Gilley J., Loreto A., Trapanotto A.G., Antoniou C., Merlini E., Coleman M.P., Orsomando G. (2022). SARM1 is a multi-functional NAD(P)ase with prominent base exchange activity, all regulated bymultiple physiologically relevant NAD metabolites. iScience.

[54] Loreto A., Di Stefano M., Gering M., Conforti L. (2015). Wallerian Degeneration Is Executed by an NMN-SARM1-Dependent Late Ca(2+) Influx but Only Modestly Influenced by Mitochondria. Cell Rep..

[55] Zhao Z.Y., Xie X.J., Li W.H., Liu J., Chen Z., Zhang B., Li T., Li S.L., Lu J.G., Zhang L. (2019). A Cell-Permeant Mimetic of NMN Activates SARM1 to Produce Cyclic ADP-Ribose and Induce Non-apoptotic Cell Death. iScience.

[56] Loreto A., Hill C.S., Hewitt V.L., Orsomando G., Angeletti C., Gilley J., Lucci C., Sanchez-Martinez A., Whitworth A.J., Conforti L. (2020). Mitochondrial impairment activates the Wallerian pathway through depletion of NMNAT2 leading to SARM1-dependent axon degeneration. Neurobiol. Dis..

[57] Summers D.W., DiAntonio A., Milbrandt J. (2014). Mitochondrial dysfunction induces Sarm1-dependent cell death in sensory neurons. J. Neurosci..

[58] Maday S. (2016). Mechanisms of neuronal homeostasis: Autophagy in the axon. Brain Res..

[59] Kim H.R., Lee H.J., Jeon Y., Jang S.Y., Shin Y.K., Yun J.H., Park H.J., Koh H., Lee K.E., Shin J.E. (2024). Targeting SARM1 improves autophagic stress-induced axonal neuropathy. Autophagy.

[60] BioRender: Scientific Illustration Software. https://BioRender.com/ypj9clj.

[61] Myers M.G., Affinati A.H., Richardson N., Schwartz M.W. (2021). Central nervous system regulation of organismal energy and glucose homeostasis. Nat. Metab..

[62] Hyun U., Sohn J.W. (2022). Autonomic control of energy balance and glucose homeostasis. Exp. Mol. Med..

[63] Wangler S., Jarczok M.N., Ennis M., Herhaus B., Wagner R., Sehgal R., Heni M. (2026). The autonomic nervous system in the regulation of glucose and lipid metabolism. Nat. Rev. Endocrinol..

[64] Amir M., Yu M., He P., Srinivasan S. (2020). Hepatic Autonomic Nervous System and Neurotrophic Factors Regulate the Pathogenesis and Progression of Non-alcoholic Fatty Liver Disease. Front. Med..

[65] Feldman E.L., Callaghan B.C., Pop-Busui R., Zochodne D.W., Wright D.E., Bennett D.L., Bril V., Russell J.W., Viswanathan V. (2019). Diabetic neuropathy. Nat. Rev. Dis. Primers.

[66] Sloan G., Selvarajah D., Tesfaye S. (2021). Pathogenesis, diagnosis and clinical management of diabetic sensorimotor peripheral neuropathy. Nat. Rev. Endocrinol..

[67] Elafros M.A., Andersen H., Bennett D.L., Savelieff M.G., Viswanathan V., Callaghan B.C., Feldman E.L. (2022). Towards prevention of diabetic peripheral neuropathy: Clinical presentation, pathogenesis, and new treatments. Lancet Neurol..

[68] Kool D., Hoeijmakers J.G., Waxman S.G., Faber C.G. (2024). Small fiber neuropathy. Int. Rev. Neurobiol..

[69] Turkiew E., Falconer D., Reed N., Höke A. (2017). Deletion of Sarm1 gene is neuroprotective in two models of peripheral neuropathy. J. Peripher. Nerv. Syst..

[70] Cheng Y., Liu J., Luan Y., Liu Z., Lai H., Zhong W., Yang Y., Yu H., Feng N., Wang H. (2019). Sarm1 Gene Deficiency Attenuates Diabetic Peripheral Neuropathy in Mice. Diabetes.

[71] Ernsberger U., Rohrer H. (2018). Sympathetic tales: Subdivisons of the autonomic nervous system and the impact of developmental studies. Neural Dev..

[72] Wehrwein E.A., Orer H.S., Barman S.M. (2016). Overview of the Anatomy, Physiology, and Pharmacology of the Autonomic Nervous System. Compr. Physiol..

[73] Liu D.S., Xu T.L. (2019). Cell-Type Identification in the Autonomic Nervous System. Neurosci. Bull..

[74] Kupari J., Häring M., Agirre E., Castelo-Branco G., Ernfors P. (2019). An Atlas of Vagal Sensory Neurons and Their Molecular Specialization. Cell Rep..

[75] Bai L., Mesgarzadeh S., Ramesh K.S., Huey E.L., Liu Y., Gray L.A., Aitken T.J., Chen Y., Beutler L.R., Ahn J.S. (2019). Genetic Identification of Vagal Sensory Neurons That Control Feeding. Cell.

[76] Zhao Q., Yu C.D., Wang R., Xu Q.J., Dai Pra R., Zhang L., Chang R.B. (2022). A multidimensional coding architecture of the vagal interoceptive system. Nature.

[77] Usoskin D., Furlan A., Islam S., Abdo H., Lönnerberg P., Lou D., Hjerling-Leffler J., Haeggström J., Kharchenko O., Kharchenko P.V. (2015). Unbiased classification of sensory neuron types by large-scale single-cell RNA sequencing. Nat. Neurosci..

[78] Nguyen M.Q., von Buchholtz L.J., Reker A.N., Ryba N.J., Davidson S. (2021). Single-nucleus transcriptomic analysis of human dorsal root ganglion neurons. eLife.

[79] Jung M., Dourado M., Maksymetz J., Jacobson A., Laufer B.I., Baca M., Foreman O., Hackos D.H., Riol-Blanco L., Kaminker J.S. (2023). Cross-species transcriptomic atlas of dorsal root ganglia reveals species-specific programs for sensory function. Nat. Commun..

[80] Li C.L., Li K.C., Wu D., Chen Y., Luo H., Zhao J.R., Wang S.S., Sun M.M., Lu Y.J., Zhong Y.Q. (2016). Somatosensory neuron types identified by high-coverage single-cell RNA-sequencing and functional heterogeneity. Cell Res..

[81] Dubin A.E., Patapoutian A. (2010). Nociceptors: The sensors of the pain pathway. J. Clin. Investig..

[82] Proske U., Gandevia S.C. (2012). The proprioceptive senses: Their roles in signaling body shape, body position and movement, and muscle force. Physiol. Rev..

[83] Abraira V.E., Ginty D.D. (2013). The sensory neurons of touch. Neuron.

[84] Mann M., West G.B. (1950). The nature of hepatic and splenic sympathin. Br. J. Pharmacol. Chemother..

[85] Klingman G.I. (1965). Catecholamine Levels and Dopa-Decarboxylase Activity in Peripheral Organs and Adrenergic Tissues in the Rat After Immunosympathectomy. J. Pharmacol. Exp. Ther..

[86] Adori C., Daraio T., Kuiper R., Barde S., Horvathova L., Yoshitake T., Ihnatko R., Valladolid-Acebes I., Vercruysse P., Wellendorf A.M. (2021). Disorganization and degeneration of liver sympathetic innervations in nonalcoholic fatty liver disease revealed by 3D imaging. Sci. Adv..

[87] Liu K., Yang L., Wang G., Liu J., Zhao X., Wang Y., Li J., Yang J. (2021). Metabolic stress drives sympathetic neuropathy within the liver. Cell Metab..

[88] Skaaring P., Bierring F. (1976). On the intrinsic innervation of normal rat liver. Histochemical and scanning electron microscopical studies. Cell Tissue Res..

[89] Sutherland S.D. (1964). An Evaluation of Cholinesterase Techniques in the Study of the Intrinsic Innervation of the Liver. J. Anat..

[90] Mikhail Y., Saleh A.L. (1961). Intrinsic nerve fibers in the liver parenchyma. Anat. Rec..

[91] Wimalawansa S.J., Emson P.C., MacIntyre I. (1987). Regional distribution of calcitonin gene-related peptide and its specific binding sites in rats with particular reference to the nervous system. Neuroendocrinology.

[92] Ito Y., Magari S., Sakanaka M. (1990). Immunoelectron-microscopic localization of peptidergic nerve fibers around lymphatic capillaries in the rat liver. Arch. Histol. Cytol..

[93] Carlei F., Lygidakis N.J., Speranza V., Brummelkamp W.H., McGurrin J.F., Pietroletti R., Lezoche E., Bostwick D.G. (1988). Neuroendocrine innervation of the hepatic vessels in the rat and in man. J. Surg. Res..

[94] el-Salhy M., Stenling R., Grimelius L. (1993). Peptidergic innervation and endocrine cells in the human liver. Scand. J. Gastroenterol..

[95] Chen C.C., Peng S.J., Chou Y.H., Lee C.Y., Lee P.H., Hu R.H., Ho M.C., Chung M.H., Hsiao F.T., Tien Y.W. (2024). Human liver afferent and efferent nerves revealed by 3-D/Airyscan super-resolution imaging. Am. J. Physiol. Endocrinol. Metab..

[96] Hwang J., Lee S., Okada J., Liu L., Pessin J.E., Chua S.C., Schwartz G.J., Jo Y.H. (2025). Liver-innervating vagal sensory neurons are indispensable for the development of hepatic steatosis and anxiety-like behavior in diet-induced obese mice. Nat. Commun..

[97] Smith R.E., Hock R.J. (1963). Brown fat: Thermogenic effector of arousal in hibernators. Science.

[98] Smith R.E., Roberts J.C. (1964). Thermogenesis of Brown Adipose Tissue in Cold-Acclimated Rats. Am. J. Physiol..

[99] Wirsén C. (1965). Distribution of adrenergic nerve fibers in brown and white adipose tissue. Compr. Physiol..

[100] Wirsén C. (1964). Adrenergic Innervation of Adipose Tissue examined by Fluorescence Microscopy. Nature.

[101] Hull D., Segall M.M. (1965). Sympathetic nervous control of brown adipose tissue and heat production in the new-born rabbit. J. Physiol..

[102] Jiang H., Ding X., Cao Y., Wang H., Zeng W. (2017). Dense Intra-adipose Sympathetic Arborizations Are Essential for Cold-Induced Beiging of Mouse White Adipose Tissue. Cell Metab..

[103] Wang P., Loh K.H., Wu M., Morgan D.A., Schneeberger M., Yu X., Chi J., Kosse C., Kim D., Rahmouni K. (2020). A leptin-BDNF pathway regulating sympathetic innervation of adipose tissue. Nature.

[104] François M., Torres H., Huesing C., Zhang R., Saurage C., Lee N., Qualls-Creekmore E., Yu S., Morrison C.D., Burk D. (2019). Sympathetic innervation of the interscapular brown adipose tissue in mouse. Ann. N. Y. Acad. Sci..

[105] Neri D., Lee S., Fohn A.M., Chen X., Bozec D., Lafond A.J., Lopatinsky N.R., Castro E.S.L., Nair G.M., Ramos-Lobo A.M. (2026). Distinct sympathetic projections to brown fat regulate thermogenesis and glucose tolerance. Nat. Metab..

[106] Sievers W., Rathner J.A., Green R.A., Kettle C., Irving H.R., Whelan D.R., Fernandez R.G.D., Zacharias A. (2020). Innervation of supraclavicular adipose tissue: A human cadaveric study. PLoS ONE.

[107] Mori S., Beyer R.S., Bernardes de Souza B., Sorg J.M., Hoover D.B., Sacks H.S., Fishbein M.C., Chang G., Peacock W.J., St John M.A. (2023). Sympathetic innervation of the supraclavicular brown adipose tissue: A detailed anatomical study. PLoS ONE.

[108] Knehans A.W., Romsos D.R. (1982). Reduced norepinephrine turnover in brown adipose tissue of ob/ob mice. Am. J. Physiol..

[109] Yoshida T., Nishioka H., Nakamura Y., Kanatsuna T., Kondo M. (1985). Reduced norepinephrine turnover in brown adipose tissue of pre-obese mice treated with monosodium-L-glutamate. Life Sci..

[110] Sakaguchi T., Arase K., Fisler J.S., Bray G.A. (1989). Effect of a high-fat diet on firing rate of sympathetic nerves innervating brown adipose tissue in anesthetized rats. Physiol. Behav..

[111] Sanchez-Rangel E., Gallezot J.D., Yeckel C.W., Lam W., Belfort-DeAguiar R., Chen M.K., Carson R.E., Sherwin R., Hwang J.J. (2020). Norepinephrine transporter availability in brown fat is reduced in obesity: A human PET study with [(11)C] MRB. Int. J. Obes..

[112] Wolf Y., Boura-Halfon S., Cortese N., Haimon Z., Sar Shalom H., Kuperman Y., Kalchenko V., Brandis A., David E., Segal-Hayoun Y. (2017). Brown-adipose-tissue macrophages control tissue innervation and homeostatic energy expenditure. Nat. Immunol..

[113] Sakamoto T., Nitta T., Maruno K., Yeh Y.S., Kuwata H., Tomita K., Goto T., Takahashi N., Kawada T. (2016). Macrophage infiltration into obese adipose tissues suppresses the induction of UCP1 level in mice. Am. J. Physiol. Endocrinol. Metab..

[114] Omran F., Christian M. (2020). Inflammatory Signaling and Brown Fat Activity. Front. Endocrinol..

[115] Villarroya F., Cereijo R., Villarroya J., Gavaldà-Navarro A., Giralt M. (2018). Toward an Understanding of How Immune Cells Control Brown and Beige Adipobiology. Cell Metab..

[116] Giordano A., Frontini A., Castellucci M., Cinti S. (2004). Presence and distribution of cholinergic nerves in rat mediastinal brown adipose tissue. J. Histochem. Cytochem..

[117] Wei H., Chiba S., Moriwaki C., Kitamura H., Ina K., Aosa T., Tomonari K., Gotoh K., Masaki T., Katsuragi I. (2015). A clinical approach to brown adipose tissue in the para-aortic area of the human thorax. PLoS ONE.

[118] De Matteis R., Ricquier D., Cinti S. (1998). TH-, NPY-, SP-, and CGRP-immunoreactive nerves in interscapular brown adipose tissue of adult rats acclimated at different temperatures: An immunohistochemical study. J. Neurocytol..

[119] Bartness T.J., Vaughan C.H., Song C.K. (2010). Sympathetic and sensory innervation of brown adipose tissue. Int. J. Obes..

[120] Norman D., Mukherjee S., Symons D., Jung R.T., Lever J.D. (1988). Neuropeptides in interscapular and perirenal brown adipose tissue in the rat: A plurality of innervation. J. Neurocytol..

[121] Lai M., Zhou W., Zou W., Qiu L., Liang Z., Chen W., Wang Y., Guo B., Zhao C., Zhang S. (2025). Brown adipose tissue secretes OLFM4 to coordinate sensory and sympathetic innervation via Schwann cells. Nat. Commun..

[122] Correll J.W. (1963). Adipose tissue: Ability to respond to nerve stimulation in vitro. Science.

[123] Diculescu I., Stoica M. (1970). Fluorescence histochemical investigation on the adrenergic innervation of the white adipose tissue in the rat. J. Neurovisc. Relat..

[124] Slavin B.G., Ballard K.W. (1978). Morphological studies on the adrenergic innervation of white adipose tissue. Anat. Rec..

[125] Vitali A., Murano I., Zingaretti M.C., Frontini A., Ricquier D., Cinti S. (2012). The adipose organ of obesity-prone C57BL/6J mice is composed of mixed white and brown adipocytes. J. Lipid Res..

[126] Bartness T.J., Song C.K. (2007). Thematic review series: Adipocyte biology. Sympathetic and sensory innervation of white adipose tissue. J. Lipid Res..

[127] Bartness T.J., Liu Y., Shrestha Y.B., Ryu V. (2014). Neural innervation of white adipose tissue and the control of lipolysis. Front. Neuroendocrinol..

[128] Chi J., Wu Z., Choi C.H.J., Nguyen L., Tegegne S., Ackerman S.E., Crane A., Marchildon F., Tessier-Lavigne M., Cohen P. (2018). Three-Dimensional Adipose Tissue Imaging Reveals Regional Variation in Beige Fat Biogenesis and PRDM16-Dependent Sympathetic Neurite Density. Cell Metab..

[129] Meng X., Qian X., Ding X., Wang W., Yin X., Zhuang G., Zeng W. (2022). Eosinophils regulate intra-adipose axonal plasticity. Proc. Natl. Acad. Sci. USA.

[130] Perdikari A., Cacciottolo T., Henning E., Mendes de Oliveira E., Keogh J.M., Farooqi I.S. (2022). Visualization of sympathetic neural innervation in human white adipose tissue. Open Biol..

[131] Cao Y., Wang H., Zeng W. (2018). Whole-tissue 3D imaging reveals intra-adipose sympathetic plasticity regulated by NGF-TrkA signal in cold-induced beiging. Protein Cell.

[132] Blaszkiewicz M., Willows J.W., Dubois A.L., Waible S., DiBello K., Lyons L.L., Johnson C.P., Paradie E., Banks N., Motyl K. (2019). Neuropathy and neural plasticity in the subcutaneous white adipose depot. PLoS ONE.

[133] Zeng W., Pirzgalska R.M., Pereira M.M., Kubasova N., Barateiro A., Seixas E., Lu Y.H., Kozlova A., Voss H., Martins G.G. (2015). Sympathetic neuro-adipose connections mediate leptin-driven lipolysis. Cell.

[134] Giordano A., Song C.K., Bowers R.R., Ehlen J.C., Frontini A., Cinti S., Bartness T.J. (2006). White adipose tissue lacks significant vagal innervation and immunohistochemical evidence of parasympathetic innervation. Am. J. Physiol. Regul. Integr. Comp. Physiol..

[135] Berthoud H.R., Fox E.A., Neuhuber W.L. (2006). Vagaries of adipose tissue innervation. Am. J. Physiol. Regul. Integr. Comp. Physiol..

[136] Severi I., Perugini J., Ruocco C., Coppi L., Pedretti S., Di Mercurio E., Senzacqua M., Ragni M., Imperato G., Valerio A. (2024). Activation of a non-neuronal cholinergic system in visceral white adipose tissue of obese mice and humans. Mol. Metab..

[137] Ballantyne B. (1968). Histochemical and biochemical aspects of cholinesterase activity of adipose tissue. Arch. Int. Pharmacodyn. Ther..

[138] Kreier F., Fliers E., Voshol P.J., Van Eden C.G., Havekes L.M., Kalsbeek A., Van Heijningen C.L., Sluiter A.A., Mettenleiter T.C., Romijn J.A. (2002). Selective parasympathetic innervation of subcutaneous and intra-abdominal fat–functional implications. J. Clin. Investig..

[139] Shi H., Song C.K., Giordano A., Cinti S., Bartness T.J. (2005). Sensory or sympathetic white adipose tissue denervation differentially affects depot growth and cellularity. Am. J. Physiol. Regul. Integr. Comp. Physiol..

[140] Wang Y., Zhang Y., Leung V.H., Seradj S.H., Sonmez U., Servin-Vences M.R., Xiao S., Ren X., Wang L., Mishkanian S.A. (2025). A key role of PIEZO2 mechanosensitive ion channel in adipose sensory innervation. Cell Metab..

[141] Passini F.S., Bornstein B., Rubin S., Kuperman Y., Krief S., Masschelein E., Mehlman T., Brandis A., Addadi Y., Shalom S.H. (2025). Piezo2 in sensory neurons regulates systemic and adipose tissue metabolism. Cell Metab..

[142] Wang Y., Leung V.H., Zhang Y., Nudell V.S., Loud M., Servin-Vences M.R., Yang D., Wang K., Moya-Garzon M.D., Li V.L. (2022). The role of somatosensory innervation of adipose tissues. Nature.

[143] Sun Y., Wang Q., Wang Y., Ren W., Cao Y., Li J., Zhou X., Fu W., Yang J. (2021). Sarm1-mediated neurodegeneration within the enteric nervous system protects against local inflammation of the colon. Protein Cell.

[144] Zhu Y., Yao L., Gallo-Ferraz A.L., Bombassaro B., Simões M.R., Abe I., Chen J., Sarker G., Ciccarelli A., Zhou L. (2024). Sympathetic neuropeptide Y protects from obesity by sustaining thermogenic fat. Nature.

[145] Mundinger T.O., Mei Q., Figlewicz D.P., Lernmark A., Taborsky G.J. (2003). Impaired glucagon response to sympathetic nerve stimulation in the BB diabetic rat: Effect of early sympathetic islet neuropathy. Am. J. Physiol. Endocrinol. Metab..

[146] Taborsky G.J., Mei Q., Hackney D.J., Figlewicz D.P., LeBoeuf R., Mundinger T.O. (2009). Loss of islet sympathetic nerves and impairment of glucagon secretion in the NOD mouse: Relationship to invasive insulitis. Diabetologia.

[147] Kraft G., Vrba A., Scott M., Allen E., Edgerton D.S., Williams P.E., Vafai S.B., Azamian B.R., Cherrington A.D. (2019). Sympathetic Denervation of the Common Hepatic Artery Lessens Glucose Intolerance in the Fat- and Fructose-Fed Dog. Diabetes.

[148] Hughes R.O., Bosanac T., Mao X., Engber T.M., DiAntonio A., Milbrandt J., Devraj R., Krauss R. (2021). Small Molecule SARM1 Inhibitors Recapitulate the SARM1(-/-) Phenotype and Allow Recovery of a Metastable Pool of Axons Fated to Degenerate. Cell Rep..

[149] Bosanac T., Hughes R.O., Engber T., Devraj R., Brearley A., Danker K., Young K., Kopatz J., Hermann M., Berthemy A. (2021). Pharmacological SARM1 inhibition protects axon structure and function in paclitaxel-induced peripheral neuropathy. Brain.

[150] Feldman H.C., Merlini E., Guijas C., DeMeester K.E., Njomen E., Kozina E.M., Yokoyama M., Vinogradova E., Reardon H.T., Melillo B. (2022). Selective inhibitors of SARM1 targeting an allosteric cysteine in the autoregulatory ARM domain. Proc. Natl. Acad. Sci. USA.

[151] Zhang W., Zhou Q., Zhang J., Wang J., Wu Q., Zheng S., Wang X. (2026). SARM1 activation promotes axonal degeneration via a two-step phase transition. Nat. Chem. Biol..

[152] Horne R.I., Andrzejewska E.A., Alam P., Brotzakis Z.F., Srivastava A., Aubert A., Nowinska M., Gregory R.C., Staats R., Possenti A. (2024). Discovery of potent inhibitors of α-synuclein aggregation using structure-based iterative learning. Nat. Chem. Biol..

[153] Ekins S., Lane T.R. (2025). Applications of artificial intelligence in drug discovery for neurological diseases. Neurotherapeutics.

